# Uropathogenic Microorganisms After Pelvic Floor Surgery and Mid-Urethral Sling Application: Detection Under Different Culture Conditions

**DOI:** 10.7759/cureus.95145

**Published:** 2025-10-22

**Authors:** Georgios Balaouras, Polychronis Kostoulas, Vassiliki Koulourida, Paraskevas Ioannidis, Iakovos Theodoulidis, Eleni Siskou, Artemis Kolynou, Maria Paraskeva, Dimitrios Balaouras, Olympia Lioupi, Dimitrios Chitzios, Themistoklis Mikos

**Affiliations:** 1 1st Department of Obstetrics &amp; Gynecology, Papageorgiou General Hospital, Aristotle University of Thessaloniki, Thessaloniki, GRC; 2 School of Health Sciences, Faculty of Public &amp; One Health, University of Thessaly, Larissa, GRC; 3 Biopathology Laboratory, Papageorgiou General Hospital, Thessaloniki, GRC; 4 1st Department of Obstetrics and Gynecology, Papageorgiou General Hospital of Thessaloniki, Thessaloniki, GRC; 5 Midwifery Department, International Hellenic University, Thessaloniki, GRC; 6 2nd Department of Obstetrics and Gynaecology, Aristotle University of Thessaloniki, Thessaloniki, GRC; 7 Obstetrics and Gynaecology Department, Aristotle University of Thessaloniki, Thessaloniki, GRC

**Keywords:** enhanced quantitative urine culture (equc), mid-urethral slings (mus), standard urine culture, urinary incontinence, urinary tract infection (uti)

## Abstract

Urinary incontinence (UI) is a prevalent condition that significantly affects quality of life and healthcare systems. Stress urinary incontinence (SUI) is often treated with mid-urethral sling (MUS), a procedure associated with a risk of postoperative urinary tract infections.

This study aimed to evaluate the effectiveness of enhanced quantitative urine culture (EQUC) for detecting uropathogens after MUS compared to conventional culture techniques. In total, 104 women of reproductive age and postmenopausal status were included. Thirty-five women with UI underwent MUS (cases), while 69 undergoing other pelvic floor reconstructions served as controls. Catheterized urine samples were collected preoperatively for both groups and postoperatively only for cases, yielding a total of 139 samples. Each sample was cultured using both conventional and EQUC methods.

EQUC detected 66.6% (12 vs. 8 positives) more uropathogens than conventional culture. McNemar’s exact test on paired results showed that this difference was not statistically significant in the two-sided test (p = 0.125), but the one-sided test suggested a trend toward higher sensitivity of EQUC (p = 0.063). Notably, no pathogens identified by conventional culture were missed by EQUC, supporting its potential as a more comprehensive diagnostic technique. Key uropathogens identified included *Escherichia coli*, *Klebsiella pneumoniae*, *Enterobacter cloacae*, and *Enterococcus faecalis*. The antibiotic sensitivity profiles reveal variable resistance patterns in detected uropathogens, especially in *Escherichia coli* and *Klebsiella pneumoniae*, highlighting the importance of targeted antibiotic therapies for MUS cases.

Although limited by sample size and single-center design, these findings indicate a promising but not statistically significant trend, which suggests that EQUC may provide clinically meaningful advantages over standard culture. Adoption of EQUC in postoperative urinary diagnostics could improve pathogen detection, enable more targeted antibiotic therapy, and reduce complications in women undergoing MUS procedures. Our results warrant validation in larger, multicenter studies.

## Introduction

Urinary incontinence (UI), defined as any complaint of involuntary loss of urine, is a prevalent and clinically significant condition [1-2] that affects millions worldwide, leading to substantial impacts on quality of life and health systems [3]. The World Health Organization recognizes UI as an international health problem [4], which affects approximately 17% of women aged ≥20 years and over 30-40% of elderly women [5-6]. Nearly half of all women who have given birth will experience some form of incontinence during their lifetime [7]. Furthermore, stress urinary incontinence (SUI) is the most common subtype with considerable economic and psychosocial burdens [8], while urge UI, often linked to detrusor overactivity, and mixed or overflow types account for additional cases [9]. Since the introduction of the retropubic tension-free vaginal tape (TVT) in 1996 [10], the mid-urethral sling (MUS) has become the gold standard surgical treatment for SUI and stress-dominant mixed incontinence. Long-term data demonstrate high subjective and objective cure rates and patient satisfaction up to five years post-surgery [11-13].

When MUS is performed, often in combination with other pelvic reconstruction procedures, the postoperative risk of urinary tract infection (UTI) increases [14-16]. Surgical-specific factors such as intraoperative catheterization, tissue trauma, and the presence of a synthetic foreign body further predispose to infection, with reported postoperative UTI rates after MUS varying widely from 7-13% in some series to as high as 30-40% within the first six weeks [17-19]. Although international guidelines recommend preoperative antibiotic prophylaxis in clean-contaminated pelvic procedures, there has been little consensus on optimal postoperative regimens, including antibiotic type, route, or duration. In a retrospective observational Greek study, *Escherichia coli* was the most frequently isolated pathogen, alongside *Klebsiella pneumoniae*, *Proteus mirabilis*, and *Enterococcus faecalis*. This indicates a diverse microbial profile of postoperative infections following MUS placement [20]. However, no studies have yet examined the optimal microbiological conditions for detecting such uropathogens. Unlike standard urine culture techniques, which use conventional media and 24-hour incubation, the enhanced quantitative urine culture (EQUC) method employs extended incubation and multiple media and could have the potential to improve the recovery of low-concentration or fastidious organisms.

The aim of this study was to evaluate whether the modification of traditional urine culture conditions and the use of EQUC enhance the detection of uropathogenic microorganisms in comparison to conventional culture techniques in women undergoing MUS surgery and pelvic floor reconstruction.

## Materials and methods

Study design and participants

This was a prospective case-control study conducted from August 2023 to September 2024 in a tertiary university hospital, with close collaboration of the Microbiology and the Urogynecology departments. Local ethics committee approval was obtained as appropriately (Protocol No 2023-B2015-271). A community control group was not included because the study was restricted, by design, to women undergoing pelvic surgery.

Inclusion criteria were (1) women with SUI who did not have clinically significant pelvic organ prolapse (POP) (the study group) and (2) women with POP who did not have USI (the control group).

Exclusion criteria were (1) age <18 years, (2) women who did not consent for any surgical intervention, (3) patients with SUI who had previous incontinence surgery, (4) patients with POP who had previous prolapse surgery, (5) women unsuitable for regional/general anesthesia, and (6) women with history of recurrent UTI. Controls were age-matched at recruitment.

A total of 104 women, both of reproductive age and postmenopausal, participated in the study. Specifically, it included 35 women with UI (cases), who underwent the application of free-tension synthetic slings (MUS), and 69 age-matched women (controls), who had undergone pelvic floor reconstruction surgery (PFRS), including anterior and posterior colporrhaphy, as well as vaginal hysterectomy. Sample size reflected all consecutive eligible patients within the 13-month period.

In both the group of women, demographic data and clinical history were recorded, and the necessary clinical examinations and urodynamic testing were performed. In the control group, only demographic data and clinical history were recorded. The collection and analysis of the data were conducted in accordance with the Declaration of Helsinki of 1975.

For the women who underwent synthetic MUS tape application and PFRS other than hysterectomy, patients were discharged the day after surgery, while those who PFRS and vaginal hysterectomy were discharged after three days. In both groups of patients, a seven-day course of chemoprophylaxis (second-generation cefalosporin, or clindamycin in cases of allergies) was prescribed. This extended regimen reflects our institutional infection-control protocol for gynecologic reconstructive procedures involving the placement of foreign material. The choice of second-generation cephalosporins aligns with their broad coverage of common Gram-negative and Gram-positive uropathogens and their established efficacy in pelvic surgery prophylaxis.

**Figure 1:**
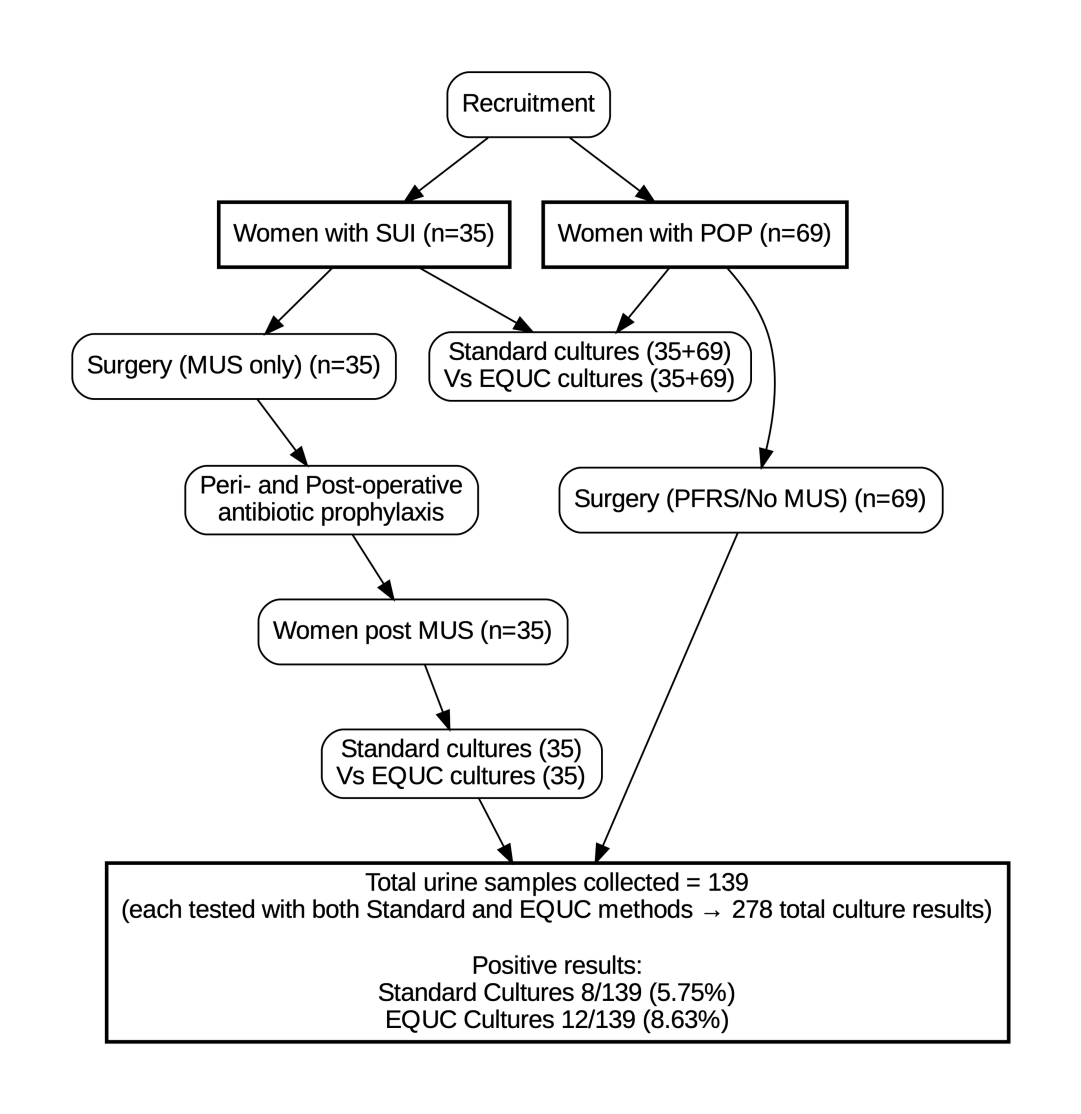
Flow diagram of participant selection, group classification, and urine sample collection. A total of 139 urine samples were collected (104 preoperative and 35 postoperative). Each sample was cultured using both Standard and EQUC methods, resulting in 278 total culture results. EQUC, expanded quantitative urine culture; MUS, mid-urethral sling; POP, pelvic organ prolapse; PFRS, pelvic floor reconstructive surgery; SUI, stress urinary incontinence

Broad-spectrum antibiotics were used, while the selection took into account the resistance profile presented by strains isolated from nosocomial infections at the given time. According to the CLSI (Clinical and Laboratory Standards Institute), three categories are recognized to characterize the effect of the antibiotic on the microbe under examination: sensitive, intermediate sensitivity, and resistant [21]. The flow of participants through the study is illustrated in Figure 1.

Urine sample collection and culture techniques

Post-operative urine collection was standardized at one to three weeks after completion of prophylaxis. Laboratory personnel performing cultures were blinded to patient group. A urinalysis was performed (taking a catheterized urine sample and carrying out a urine culture) in each patient, as part of the regular outpatient clinic check, as well as a comparison of these results. A urine sample was taken as part of the preoperative control in both groups under observation, while a urine sample was taken again postoperatively (at the post-operative visit one to three weeks after the completion of the post-operative prophylactic treatment) only for the study group (cases). All urine samples were transported immediately to the microbiology laboratory and processed within 1 hour of collection. In total, 139 urine samples were collected: 104 preoperative (one per patient) and 35 postoperative from the MUS group. Each sample was processed with both Standard and EQUC methods, yielding 278 total culture results.

Urine culture is the criterion standard for the diagnosis of UTI. The quantitative urine culture consists of inoculating 1 μL of urine using a calibrated loop or 5 μL of urine, depending on the diameter of the loop, into a citrate lactose electrolyte deficient (CLED) agar plate. Selective and differential mediums, such as MacConkey agar and Columbia colistin-nalidixic agar (CNA), can be used also. After 24-hour incubation at 35°C, the number of colonies is counted in order to calculate the number of microorganisms per milliliter.

In this study, a “positive” urine culture was defined as ≥10³ CFU/mL, consistent with the Infectious Diseases Society of America (IDSA) and European Association of Urology (EAU) recommendations for catheterized urine specimens in symptomatic patients. Urine cultures were classified as “negative” if they yielded growth of a uropathogen(s) at a count <10^3^ CFU/mL, or showed mixed growth of normal flora (staphylococci, or streptococci etc.).

All participants provided catheterized urine samples following a standardized collection protocol to minimize contamination. Samples were promptly processed and cultured using both conventional and enhanced urine culture techniques. The conventional method involved plating 1 μL of urine on blood agar plates (BAPs) and MacConkey agar, followed by aerobic incubation at 35°C for 24 hours.

For enhanced detection, a more comprehensive set of conditions (EQUC) was utilized. This included plating (a) 1 μL of urine on BAPs (BAP) and MacConkey agar, followed by incubation at 35°C, in 5% CO_2_, for 24 hours at first and for extra 24 hours, if bacterial growth was observed, and (b) 100 μL of urine on BAP, chocolate agar, and CNA agar, incubated in 5% CO_2_ at 35°C for 48 hours.

Microbial identification

Bacterial colonies were quantified and identified using standard microbiological techniques, including matrix-assisted laser desorption/ionization time-of-flight mass spectrometry (MALDI-TOF MS). Only clinically relevant uropathogens were included in the analysis.

Statistical analysis

Data were analyzed using descriptive statistics to compare outcomes between the two groups. Because of the exploratory sample size, formal multivariable adjustment was not feasible. For paired comparison of culture methods (standard vs. EQUC), McNemar’s exact test was used. Both two-sided and one-sided tests were performed: the two-sided p-value assessed overall discordance between methods, while the one-sided test specifically evaluated whether EQUC detected significantly more positives than the standard method, reflecting its hypothesized higher sensitivity. P-values <0.05 were considered statistically significant. Analyses were performed in R (version 4.4.0, R Foundation for Statistical Computing, Vienna, Austria).

## Results

A total of 104 Caucasian women were consecutively enrolled in the study. Baseline characteristics are summarized in Table 1.

**Table 1:** Demographic and baseline characteristics of the study population. Continuous variables are presented as mean ± standard deviation. Categorical variables are presented as counts and percentages. MUS, mid-urethral sling; POP, pelvic organ prolapse

Variable	Study Group (MUS, n=35)	Control Group (POP, n=69)
Mean age (years)	62.2 ± 11.3	65.7 ± 10.1
Mean BMI (kg/m²)	29.5 ± 5.4	27.5 ± 4.8
Mean parity (n)	2.3 ± 0.8	2.4 ± 0.9
Postmenopausal, n (%)	6 (16.7%)	5 (7.2%)

In the study group, all patients had SUI or mixed UI, whereas in the control group, no patient had SUI. All patients in the study group underwent MUS placement: 31 had a mini-sling under local anesthesia, 3 had a trans-obturator tape under local anesthesia, and 1 had a retropubic tape under regional anesthesia. In the control group, all women underwent prolapse surgery without any anti-incontinence procedure: 18 (26.1%) had anterior-posterior colporrhaphy only, 33 (47.8%) had vaginal hysterectomy and anterior-posterior colporrhaphy, 7 (10.1%) had sacrospinous ligament fixation of the vaginal vault and anterior-posterior colporrhaphy, and 5 (7.2%) had colpocleisis.

The results from the cases group are presented below, separated into both preoperative and postoperative findings across conventional and enhanced culture conditions.

Preoperative findings

In the cases group, six positive samples were identified, four of which were preoperative. Specifically, three samples showed positive results across all conditions, both conventional and EQUC. These included two *Escherichia col*i isolates and one *Klebsiella pneumoniae* isolate. All of them, with bacterial growth at a count >10^5^ CFU/mL of urine, while one sample was positive only under EQUC conditions. This was a *Micrococcus luteus* isolate, with bacterial growth equal to 10^3^ CFU/mL.

Postoperative findings

Additionally, two postoperative samples were positive under both conventional and EQUC conditions: one *Enterobacter cloacae* isolate and one *Klebsiella pneumoniae* isolate, both with bacterial growth at a count >10^5^ CFU/mL.

Similarly, in the control group, six positive samples were identified. Three of these samples were positive under all conditions, both conventional and EQUC, including two (2) *Escherichia coli* isolates and one (1) *Enterococcus faecalis* isolate. All of them had bacterial growth at a count >10^5^ CFU/mL. Additionally, three samples were positive only under EQUC conditions, comprising two *Enterococcus faecalis* isolates, with bacterial growth at a count >10^5^ CFU/mL and one *Bacillus simplex* isolate, with bacterial growth equal to 10^3^ CFU/mL.

The microorganisms detected in this study were all clinically relevant, as the women reported symptomatic UTIs. These pathogens are considered opportunistic, meaning they can cause infections when the body’s defenses are compromised, such as after surgical procedures such as MUS. Even the patients in whom *Micrococcus luteus* and *Bacillus simplex* isolates were detected manifested clinical symptoms of UTI and had a positive urine test preoperatively (WBCs=63/μL and WBCs=742/μL, NR: 0-25/μL, respectively). The detection of these opportunistic pathogens underscores the importance of accurate microbial identification, as these organisms, although not always harmful, can lead to clinically significant infections in susceptible individuals, particularly in a postoperative setting. This highlights the need for thorough pathogen evaluation to ensure appropriate treatment and management of UTIs in these patients.

The microorganisms identified in both the cases and control groups, as mentioned above, included *Escherichia coli*, *Klebsiella pneumoniae*, *Enterobacter cloacae*, *Enterococcus faecalis*, *Micrococcus luteus*, and *Bacillus simplex* (Table 2), most of which are known to be clinically relevant uropathogens, whereas *Micrococcus luteus* and *Bacillus simplex *are considered opportunistic or environmental species. Their detection underscores the sensitivity of the EQUC method, yet their pathogenic role in postoperative urinary infections should be interpreted cautiously.

**Table 2:** Distribution of uropathogens detected in study and control groups. Both conventional urine culture and EQUC methods were used. All identified microorganisms were considered clinically relevant based on patient-reported UTI symptoms and positive urine leukocyte counts. EQUC, enhanced quantitative urine culture; MUS, mid-urethral sling; POP, pelvic organ prolapse

Pathogen	Study Group (MUS, n=35)	Control Group (POP, n=69)	Total (n=104)
Escherichia coli	2 (5.7%)	2 (2.9%)	4 (3.8%)
Klebsiella pneumoniae	2 (5.7%)	–	2 (1.9%)
Enterobacter cloacae	1 (2.9%)	–	1 (1.0%)
Enterococcus faecalis	–	3 (4.3%)	3 (2.9%)
Micrococcus luteus	1 (2.9%)	–	1 (1.0%)
Bacillus simplex	–	1 (1.4%)	1 (1.0%)
Total positive samples	6 (17.1%)	6 (8.7%)	12 (11.5%)

More specifically, the uropathogenic microorganisms identified in this study included *Escherichia coli*, *Klebsiella pneumoniae*, *Enterobacter cloacae*, *Enterococcus faecalis*, *Micrococcus luteus*, and *Bacillus simplex*:

*Escherichia coli *was identified under all conditions in both the case and control groups (four samples, all preoperatively, >10^5^ CFU/mL). It is a Gram-negative bacterium that colonizes the gastrointestinal tract. *Escherichia coli *is the most common cause of UTIs, leading to symptoms such as dysuria, urinary frequency, and, in severe cases, pyelonephritis.

*Klebsiella pneumoniae* was identified in the case group under all conditions (two samples, one preoperatively and the other postoperatively, both >10^5^ CFU/mL). It is a Gram-negative bacterium commonly found in the gastrointestinal tract and also responsible for UTIs [22-23]. It is frequently associated with hospital-acquired infections and is known for its multidrug resistance, complicating treatment efforts [24].

*Enterobacter cloacae* was identified in the case group under all conditions (one postoperative sample, >10^5^ CFU/mL). It is a Gram-negative bacterium commonly found in the gastrointestinal tract and a known causative agent of UTIs [25]. It is frequently associated with nosocomial infections, particularly in catheterized patients, and exhibits significant antibiotic resistance, complicating treatment [26].

*Enterococcus faecalis *was identified in the control group (three samples, >10^5^ CFU/mL). One sample was detected under all conditions, while the other two were identified after the inoculation of 100 μL of urine on MacConkey agar, blood agar, and CNA agar, incubated at 35°C in 5% CO₂ for 48 hours, as part of the enhanced urine culture protocol (EQUC). This Gram-positive bacterium, commonly residing in the gastrointestinal tract, is frequently associated with UTIs [27]. It poses significant challenges due to its antibiotic resistance, including resistance to vancomycin [28].

*Micrococcus luteus *was identified in the case group (one sample, 10^3^ CFU/mL) after the inoculation of 100 μL of urine on MacConkey agar, blood agar, and CNA agar, incubated at 35°C in 5% CO₂ for 48 hours, as part of the enhanced urine culture protocol (EQUC). Micrococcus luteus is a Gram-positive coccus of the Micrococcaceae family, widely distributed in the environment, including soil, air, water, and animals. It can also be found on human skin and mucous membranes [29]. Although *Micrococcus luteus* is rarely reported in clinical cases, it has been known to affect vulnerable patient groups, such as malnourished individuals and those with weakened immune systems [30].

*Bacillus simplex* was identified in the control group (one sample, 10^3^ CFU/mL) following the inoculation of 1 μL of urine on MacConkey agar and blood agar, incubated at 35°C in 5% CO₂ for 24 hours, with an additional 24-hour incubation (48 hours total), as part of the enhanced urine culture protocol (EQUC). This Gram-positive, spore-forming, rod-shaped bacterium is primarily found in the environment. Bacillus species are aerobic or facultatively anaerobic and, while typically environmental organisms, can cause opportunistic infections under certain conditions, particularly in immunocompromised individuals [31]. Although rare, *Bacillus simplex* may lead to UTIs in susceptible patients. To further examine the effectiveness of antibiotic treatments and the prevalence of resistance among detected uropathogens, Table 3 provides a comparative summary of antibiotic treatments and the corresponding sensitivity profiles for pathogens identified in MUS patients and control participants. This table highlights the antibiotics used, treatment timing, and sensitivity or resistance patterns observed, facilitating an analysis of resistance trends in each group. By distinguishing preoperative and postoperative phases, these data help illustrate the influence of MUS procedures on microbial sensitivity and resistance, offering essential insights for targeted antibiotic management. These results underscore the variability in resistance patterns and the necessity of selecting appropriate antibiotics, as further explored in the pathogen-specific analysis below.

**Table 3:** Comparative overview of antibiotic treatments administered to MUS patients and control group participants, detailing antibiotics used, timing of administration (preoperative or postoperative), and detected uropathogens. Sensitivity profiles are represented as follows: “S” (sensitive), “R” (resistant), “I” (intermediate), and “IE” (insufficient evidence). These data allow for a comparative analysis of microbial sensitivity and resistance between MUS cases and controls, highlighting patterns in antibiotic efficacy across commonly detected pathogens such as *Escherichia coli* and *Enterococcus faecalis*. C1–C5 represent samples from control group participants, and P1–P3 represent samples from the study group (MUS patients). Pre-op. and Post-op. refer to the timing of urine sample collection (preoperative or postoperative). SYN-R indicates synergistic resistance, and SYN-S indicates synergistic sensitivity. Empty cells are marked with a dash (“–”) to indicate “not tested” due to lack of clinical relevance, limited sample volume, or intrinsic resistance.

Pathogen/Antibiotic	C1	C2	C3	C4	C5	P1	P2	P3	P3	P1
						Pre-op.	Pre-op.	Pre-op.	Post-op.	Post-op.
	Enterococcus faecalis			Escherichia coli				Klebsiella pneumoniae		Enterobacter cloacae
Ampicillin/Sulbactam	S	S	S	R	S	R	S	R	-	R
Amoxycillin/Clavulanic	S	S	-	-	-	-	-	R	R	-
Amikacin	R	R	-	S	S	S	S	R	R	S
Aztreonam	-	-	-	S	S	S	S	R	-	S
Cephalothin	-	-	R	-	-	-	-	-	-	-
Cefazolin	R	R	-	-	-	-	-	-	-	-
Cefepime	-	-	-	S	S	S	S	R	R	S
Cefixime	-	-	-	-	-	-	-	R	-	-
Cefotaxime	-	-	-	-	-	-	-	R	R	-
Cefoxitin	-	-	-	S	S	S	S	R	R	R
Ceftazidime	-	-	-	S	S	S	S	R	R	S
Ceftazidime/Avibactam	-	-	-	-	-	-	-	S	S	-
Ceftolozane/Tazobactam	-	-	-	-	-	-	-	R	R	-
Ceftriaxone	-	-	-	S	S	S	S	R	-	S
Chloramphenicol	-	-	-	-	-	-	-	S	-	-
Ciprofloxacin	R	R	S	S	S	R	S	R	R	S
Cinopristin/Dalfopristin	R	R	-	-	-	-	-	-	-	-
Clindamycin	-	-	R	-	-	-	-	-	-	-
Colistin	-	-	-	S	S	S	S	S	S	S
Daptomycin	S	S	S	-	-	-	-	-	-	-
Ertapenem	-	-	-	-	-	-	-	R	R	-
Erythromycin	-	-	I	-	-	-	-	-	-	-
Fosfomycin	-	-	-	S	S	R	S	R	R	S
Imipenem	I	I	S	S	S	S	S	R	-	S
Gentamycin	-	-		S	S	S	S	S	S	S
Gentamycin-500	SYN-R	SYN-R	S	-	-	-	-	-	-	-
Levofloxacin	-	-	-	S	S	R	S	R	-	S
Linezolid	S	S	I	-	-	-	-	-	-	-
Meropenem	-	-	-	S	S	S	S	R	R	S
Minicycline	-	-	-	-	-	-	-	-	-	-
Moxifloxacin	-	-	S	-	-	-	-	R	-	-
Nitrofurantoin	-	-	-	-	-	-	-	I	I	-
Piperacillin/Tazobactam	-	-	-	S	S	S	S	R	R	S
Streptomycin 2000	SYN-S	SYN-S	S	-	-	-	-	-	-	-
Teicoplanin	S	S	S	-	-	-	-	-	-	-
Tetracycline	-	-	R	-	-	-	-	-	-	-
Tigecycline	S	S	S	S	S	S	S	ΙΕ	IE	IE
Tobramycin	-	-	-	-	-	-	-	R	-	-
Trimethorpim/Sulfamethoxazole	-	-	R	R	S	S	S	R	R	S
Vancomycin	S	S	S	-	-	-	-	-	-	-

These resistance profiles (Table 3) reveal that *Klebsiella pneumoniae* and *Enterobacter cloacae* exhibited multidrug-resistant patterns against several β-lactams and fluoroquinolones, while maintaining sensitivity to carbapenems and colistin, while *Escherichia coli* isolates demonstrated more variable resistance, particularly to ciprofloxacin and trimethoprim-sulfamethoxazole. These patterns are consistent with globally recognized nosocomial multidrug-resistant lineages and emphasize the clinical need for postoperative antimicrobial stewardship and culture-guided therapy following MUS procedures. These findings are clinically relevant, as they highlight the need for individualized antibiotic therapy based on culture results rather than empirical postoperative regimens.

To further evaluate differences between culture methods, a paired analysis was performed across all 139 urine samples. Considering all 139 paired urine samples, standard culture detected eight (5.75%) positives and EQUC detected 12 (8.63%) positives. The paired cross-classification is shown in Table 4. Eight samples were positive by both methods, four were positive only by EQUC, and none were positive only by standard culture (127 were negative by both). Notably, no samples were positive on standard culture but negative on EQUC, consistent with the broader conditions of EQUC. McNemar’s exact test on the discordant pairs indicated a non-significant trend toward higher sensitivity of EQUC (two-sided p = 0.125; one-sided p = 0.063).

**Table 4:** Paired comparison of standard vs. EQUC urine culture results (n = 139 samples). McNemar’s exact test on the discordant pairs (4 EQUC+/standard– vs. 0 standard+/EQUC–) indicated a non-significant trend toward higher sensitivity of EQUC (two-sided p = 0.125; one-sided p = 0.063). EQUC, enhanced quantitative urine culture

	EQUC +	EQUC –	Total	p-Value
Standard +	8	0	8	-
Standard –	4	127	131	-
Total	12	127	139	Two-sided: 0.125; one-sided: 0.063

Overall, the antibiotic sensitivity profiles observed in Table 3 underscore the necessity for personalized antibiotic selection in managing UTIs, especially in postoperative MUS patients, to enhance treatment efficacy and mitigate resistance development. Although Table 3 illustrates variable resistance across the isolated uropathogens, with multidrug-resistant profiles in *Klebsiella pneumoniae* and *Enterobacter cloacae*, these findings should be interpreted with caution due to the limited number of isolates per species. The observed resistance to multiple β-lactams and fluoroquinolones in these organisms aligns with their known hospital-acquired multidrug-resistant phenotypes. However, a detailed statistical or genotypic resistance analysis was beyond the scope of this work and will be presented in a forthcoming study focusing specifically on antimicrobial susceptibility trends in postoperative pelvic surgery patients.

## Discussion

The results of this study provide significant insights into the effectiveness of EQUC methods for detecting uropathogens. When comparing EQUC with standard culture, McNemar’s exact test showed that the increase in detection with EQUC was not statistically significant in the two-sided test (p = 0.125). However, the one-sided test, performed to evaluate the specific hypothesis that EQUC is more sensitive, yielded a p-value of 0.063, suggesting a trend toward superior detection by EQUC. Although under-powered, this finding supports the biological plausibility of EQUC offering enhanced sensitivity, as the expanded culture conditions are designed to detect fastidious or low-concentration organisms. Larger studies will be needed to confirm whether this trend reflects a true diagnostic advantage. In the cases group, both preoperative and postoperative infections were identified through EQUC, confirming its ability to detect clinically significant pathogens such as *Enterobacter cloacae *and *Klebsiella pneumoniae*. These pathogens are known to contribute to postoperative complications, particularly in women who have undergone MUS procedures [23,25]. Both *Klebsiella pneumoniae* and *Enterobacter cloacae* exhibited multidrug-resistant profiles in our isolates, which is consistent with their well-established role as hospital-acquired pathogens. This resistance to several β-lactams and fluoroquinolones, combined with preserved susceptibility to carbapenems and colistin, underscores the need for postoperative antimicrobial stewardship and culture-guided therapy following MUS procedures. Furthermore, no cases were observed where standard culture was positive and EQUC was negative, consistent with the expanded methodological basis of EQUC and suggesting that EQUC could encompass the diagnostic capacity of standard culture.

In both groups (cases and controls), the EQUC revealed positive results in four samples that were not detected using conventional methods. Pathogens such as *Enterococcus faecalis*, *Micrococcus luteus*, and *Bacillus simplex* were isolated only through the EQUC process, suggesting that these microorganisms, particularly when present in lower concentrations or requiring specific growth conditions, may be missed with traditional culture techniques. This highlights the limitations of conventional methods and underscores the importance of adopting more comprehensive diagnostic approaches such as EQUC. The detection of low-concentration pathogens such as *Micrococcus luteus* and *Bacillus simplex* is clinically significant, particularly in postoperative or immunocompromised patients. While these microorganisms are often considered part of the normal flora or environmental contaminants, their presence in catheterized urine samples and their association with elevated leukocyte counts suggest a potential role in postoperative UTIs. It should also be noted that the broader sensitivity of EQUC inherently increases the likelihood of detecting environmental or commensal organisms. Hence, distinguishing true pathogens from contaminants remains an important consideration for clinical application.

Specifically, *Micrococcus luteus*, although rarely pathogenic, has been reported in immunosuppressed individuals and malnourished patients, where it can cause opportunistic infections. Similarly, *Bacillus simplex*, typically an environmental organism, may lead to clinically significant infections in vulnerable populations. Their detection underscores the importance of advanced diagnostic methods such as EQUC, which are capable of identifying these pathogens in scenarios where traditional methods might miss them.

This capability is crucial in tailoring appropriate antimicrobial therapies, as low-concentration pathogens might contribute to persistent or recurrent infections if left untreated. Moreover, understanding the clinical relevance of such organisms can help refine postoperative management protocols, minimizing the risk of complications. These findings further highlight the diagnostic advantages of EQUC. Specifically, the EQUC cultures isolated microorganisms more frequently than conventional cultures, with a higher frequency observed among controls (3/69) compared to cases (1/70). Although this finding was not statistically significant, it underscores EQUC’s enhanced sensitivity, particularly in detecting pathogens present in lower bacterial loads or requiring specific growth conditions. This capability is crucial for ensuring accurate diagnosis and optimizing clinical management in challenging cases.

The increased pathogen detection in both groups, particularly the control group, emphasizes the potential for underdiagnosis when relying solely on conventional cultures. The ability to identify additional uropathogens has critical implications for improving diagnostic accuracy and ensuring appropriate clinical management. In particular, EQUC proves vital in detecting opportunistic pathogens that may not always be pathogenic in healthy individuals but can cause significant infections in postoperative patients, where immune defenses are often compromised. No association was found between the presence of uropathogens (detected by either method) and preoperative or postoperative clinical symptoms, including stress incontinence, overactive bladder symptoms, or postoperative tape erosion.

Moreover, this study is pioneering in Greece, as it introduces the use of varied culture conditions that have not previously been applied. Until now, standard urine culture methods in Greece have consistently used the same culture conditions, potentially overlooking key pathogens. By incorporating EQUC into the study design, this research marks a significant step forward in enhancing diagnostic capabilities and providing a more comprehensive understanding of UTIs in clinical settings. Furthermore, adopting more sensitive diagnostic techniques such as EQUC can be seen as a clinically and ethically relevant consideration, as it may enhance diagnostic accuracy and reduce complications through more targeted treatment. While improved detection does not automatically translate into superior outcomes, implementing more comprehensive testing aligns with good clinical practice and patient-centered care principles [32-33]. However, EQUC also presents practical challenges, including increased laboratory workload, higher costs, and the need for standardized interpretation criteria, which may currently limit its widespread clinical adoption. Timely and precise diagnosis of infections allows healthcare provides to administer targeted treatments, minimizing unnecessary antibiotic use and reducing risk of antimicrobial resistance.

This study further underscores the value of thorough culturing in postoperative patients. The detection of opportunistic pathogens in both the cases and control groups highlights the need for comprehensive microbiological assessments in patients undergoing procedures such as MUS. Although many of these pathogens may be part of the normal flora, in immunocompromised or postoperative patients, they can lead to severe UTIs. Therefore, the use of enhanced detection methods, such as EQUC, is crucial in identifying and managing these infections early. An increased prevalence of positive urine cultures (both conventional and EQUC) was observed in older women, potentially reflecting the known higher incidence of asymptomatic bacteriuria in this population. The clinical relevance of this finding remains unclear, as bacterial isolation did not correlate with clinical symptoms or pathology, suggesting that further investigation is warranted.

The link between the detected pathogens and the clinical manifestations of UTIs further supports the importance of advanced culture techniques. All detected microorganisms were clinically relevant, as the women presented with urinary symptoms consistent with infection. This association underlines the need for more detailed microbial analysis, particularly in cases where conventional methods might fail to detect low-level or fastidious pathogens, leading to underdiagnosis and, consequently, inappropriate or delayed treatment.

Given these findings, there is a clear need to revisit and improve diagnostic protocols for UTIs, particularly in postoperative patients. The integration of enhanced culture techniques such as EQUC into routine clinical practice could lead to more accurate identification of uropathogens, enabling targeted antibiotic treatments and reducing the risk of persistent or recurrent infections. This is especially relevant in surgical contexts such as MUS, where the early and precise detection of pathogens can prevent complications and improve patient outcomes. From a clinical perspective, the application of EQUC could refine several aspects of perioperative care. Preoperatively, EQUC may help identify low-level colonization that could inform tailored antibiotic prophylaxis, particularly in women with prior urinary symptoms or risk factors for infection, while, postoperatively, its use could enable earlier detection of subclinical or persistent infections and, hence, allow more precise and timely interventions. Beyond these practical implications, an additional interesting observation was that all positive EQUC cultures were found in patients with a BMI below 27, the cohort’s mean BMI. This preliminary observation may reflect hormonal or metabolic factors associated with BMI that influence susceptibility to bladder colonization. However, this remains speculative, and further studies are required to validate this hypothesis.

Despite its strengths, this single-center feasibility study has that should be acknowledged. The relatively small sample size and unequal group sizes, without lack of full matching, may limit the generalizability of the findings. Nevertheless, key demographic characteristics such as age and menopausal status were comparable between groups. Postoperative urine cultures were not obtained from the control group, thus restricting the ability to assess potential asymptomatic or delayed infections after prolapse repair without MUS placement. Furthermore, the absence of a non-surgical, community-based control group also limits comparison with baseline urinary tract colonization in the general population. Finally, the extended seven-day antibiotic prophylaxis reflects institutional infection-control policy for reconstructive pelvic surgery and may have influenced postoperative infection rates. Although EQUC demonstrated technical feasibility within a routine clinical laboratory, larger multicenter studies with broader control groups are required to validate and extend these observations.

In conclusion, this study highlights the enhanced sensitivity of the EQUC method over conventional urine culture techniques in detecting clinically relevant uropathogens [34], particularly in postoperative MUS patients. Nevertheless, as EQUC remains a resource-intensive technique, further research should evaluate its cost-effectiveness, reproducibility across laboratories, and potential impact on clinical decision-making before routine implementation. The findings demonstrate the ability of EQUC to identify uropathogens that may be missed by traditional methods, emphasizing the potential for improved diagnostic accuracy in clinical practice. The EQUC’s demonstrated ability to detect low-concentration and fastidious pathogens highlights its potential to improve diagnostic accuracy and guide targeted antimicrobial therapy, ultimately reducing postoperative complications and antibiotic resistance. Future studies with larger cohorts and diverse populations are essential to validate these findings and establish EQUC as a gold standard in uropathogen detection.

## Conclusions

The antibiotic sensitivity profiles observed underscore the variability of resistance among detected uropathogens, particularly *Escherichia coli *and *Klebsiella pneumoniae*. Such findings highlight the potential of EQUC to provide a more detailed microbiological picture than conventional methods. EQUC may improve pathogen detection and thereby inform antibiotic selection. However, further studies with larger cohorts are required to determine whether these microbiological advantages translate into clinical benefit. There is need of multicenter studies to evaluate further the feasibility, cost-effectiveness, and patient impact of EQUC-guided diagnostics in postoperative care.
